# Early Bilateral Gonadoblastoma in a Patient with Mixed Gonadal Dysgenesis (Karyotype 45,X/46,XY): Case Report and Review of Literature

**DOI:** 10.15388/Amed.2022.29.2.5

**Published:** 2022-06-29

**Authors:** Ignas Trainavičius, Darius Dasevičius, Birutė Burnytė, Robertas Kemežys, Gilvydas Verkauskas

**Affiliations:** Vilnius University, Faculty of Medicine, Vilnius, Lithuania; National Center of Pathology, Affiliate of Vilnius University Hospital Santaros Klinikos, Vilnius, Lithuania; Vilnius University, Faculty of Medicine, Institute of Biomedical Sciences, Vilnius, Lithuania; Vilnius University, Faculty of Medicine, Institute of Clinical medicine, Vilnius, Lithuania; Vilnius University, Faculty of Medicine, Vilnius, Lithuania

**Keywords:** Disorders of sex development, Gonadal dysgenesis, Mixed gonadal dysgenesis, 45,X/46,XY mosaicism, Gonadoblastoma, Gonadectomy

## Abstract

**Background:**

Mixed gonadal dysgenesis is a rare congenital and challenging condition, characterized mainly by 45,X/46,XY karyotype mosaicism, asymmetrical gonadal development and various internal and external genital anatomy. Because of frequent disorder of genital development and a higher risk of germ cell neoplasia, management of these patients is complex and requires multidisciplinary approach.

**Case:**

We present a 45,X/46,XY mixed gonadal dysgenesis patient diagnosed with gonadoblastoma in both gonads after bilateral gonadectomy at 1 year of age.

**Conclusions:**

Because of high risk for malignant transformation, gonadectomy of a streak-like gonad and biopsy with orchidopexy or gonadectomy of a dysgenetic testicle is recommended at an early age.

## Santrauka.

Mišri lytinių liaukų disgenezė yra reta įgimta ir sudėtinga būklė, kuriai būdingas 45,X/46,XY mozaikinis kariotipas, asimetrinis lytinių liaukų vystymasis ir įvairialypė vidinių ir išorinių lytinių organų anatomija. Dėl didesnės lytinių ląstelių neoplazijos rizikos ir kitų svarbių veiksnių šių pacientų gydymas yra sudėtingas ir reikia daugiadisciplininio požiūrio.

**Atvejis:**

Pristatome 45,X/46,XY mišrios lytinių liaukų disgenezės atvejį, kai po abipusės gonadektomijos Vienų metų amžiaus pacientui diagnozuota abiejų lytinių liaukų gonadoblastoma.

**Išvados:**

Dėl didelės piktybinės transformacijos rizikos pašalinti ruoželinę gonadą ir atlikti disgenetiškos sėklidės biopsiją ir nuleidimą arba pašalinimą rekomenduojama ankstyvame amžiuje.

## Case

The index patient was born with ambiguous genitalia. Clinical examination revealed posterior penoscrotal hypospadia, unpalpable gonads and bilateral inguinal hernias. Transabdominal ultrasound (US) examination revealed 2.81 cm length uterus: 1.07 cm cervix, 1.74 cm fundus and 3.0 × 1.94 cm vagina. Cardiac US revealed atrial septal defect and bicuspid aortic valve. Abdomen and pelvic magnetic resonance imaging showed normal size horseshoe kidney, anteversion of uterus, minimal hydrocolpos: dilated vagina to 1.4 cm and persistent urogenital sinus. Moreover, on the left near the uterus, a 1.0 × 0,5 cm structure was seen that resembled an ovary. On the right near the bladder a 2.0 × 0.7 cm cystic structure was visualized that connected to the uterus like a fallopian tube. Serum hormone levels presented in Table 1. Testosterone levels after birth and at the beginning of “mini puberty” (36 days) are clearly above the normal female range, but within the typical male range, reflecting normal Leydig cell function from testicular tissue. Normal gonadotropin levels suggest normal gonadal endocrine function of testicular and ovarian tissues, as confirmed later after bilateral gonadectomy. Normal or slightly elevated serum 17-hydroxyprogesterone level at birth ruled out classical congenital adrenal hyperplasia (CAH) due to 21 hydroxylase deficiency, which is considered to be the most common cause of disorders of sex development (DSD) requiring immediate therapy, because karyotype result usually comes later. According to multidisciplinary decision together with the parents, female sex of rearing was chosen.

**Table 1.** Postnatal serum sex steroid and gonadotropin levels (before and after bilateral gonadectomy).

Age	Testosterone, (nmol/L)	Estradiol, (pmol/L)	LH, (IU/L)	FSH, (IU/L)	17-OHP (nmol/L)
2 days	18.37		4.5	4.7	22
16 days	10.20				
36 days	11.46				
1 year 1 month	0.24	73.4	0.20	1.1	
1 year 3 moths	0.26		0.28	1.1	

LH – Luteinizing hormone, FSH – Follicle-stimulating hormone.

Laparoscopic gonadectomy, cystovaginoscopy and bilateral inguinal hernia repair operations were performed at the age of 1 year and 2 months. A dysmorphic gonad resembling a dysgenetic testis was removed from the left and a streak-like gonad from the right. Gonads were sent for pathological evaluation and biopsies from the dysmorphic gonad and umbilical skin sent for genetic evaluation. Cytogenetic analysis of the cultured peripheral blood samples showed high-level Turner syndrome mosaicism with presence of Y chromosome – 45,X[32]/46,XY[3]. Karyotype result of skin fibroblasts was 45,X[24]/46,XY[6]. Predominant 45,X karyotype from peripheral blood and skin fibroblasts explains Turner syndrome phenotype: short stature (patient’s height at 1 year and 3 months was lower than 3rd percentile), left side heart anomaly (bicuspid aortic valve) and horseshoe kidney. Because of short stature growth hormone therapy was planned from 4-6 years of age. Feminizing genitoplasty was performed at the age of 1 year and 11 months.

**Figure 1.**
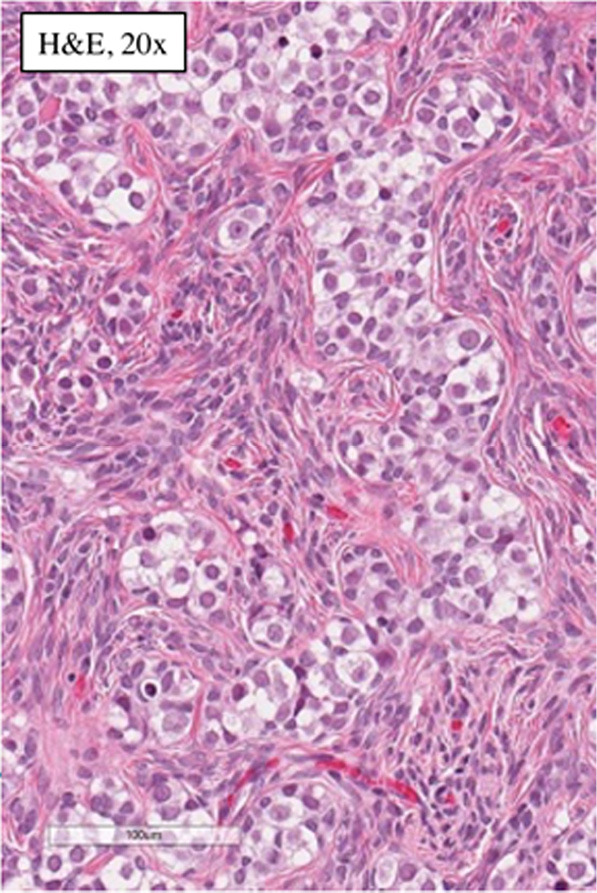
Histology of the right gonad: streak gonad with gonadoblastoma.

**Figure 2.**
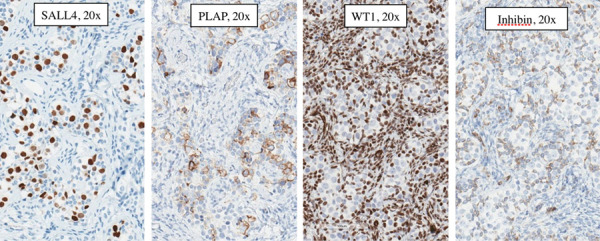
Right gonad gonadoblastoma immunohistochemistry: Germ cells: SALL4 (Spalt-like transcription factor 4) – positive, PLAP (placental-like alkaline phosphatase) – positive, WT1 (Wilms tumor 1) – negative, Inhibin – negative; Sex cord cells: SALL4 (Spalt-like transcription factor 4) – negative, PLAP (placental-like alkaline phosphatase) – negative, WT1 (Wilms tumor 1) – positive, Inhibin – positive.

Pathology of the left gonad: dysgenetic testis containing seminiferous tubules filled with Sertoli cells and a small number of spermatogonia; deficient stroma with poorly visualised Leydig cells; epididymis and fallopian tube tissue; small undifferentiated gonadal fragment with gonadoblastoma as an appendiceal outgrowth of testicular part. Pathology of the right gonad revealed streak-like gonad with gonadoblastoma and fallopian tube tissue. Tumor imunohistochemistry is shown in Table 2. Right gonad histology is shown in Figure 1, and imunohistochemistry in Figure 2.

**Table 2.** Tumor imunohistochemistry.

	Left gonad		Right gonad	
	Germ cells	Sex cord cells	Germ cells	Sex cord cells
CD117	+++, 100% (cytoplasm reaction)	negative	+++, 100% (cytoplasm reaction)	negative
D2-40	+++, 100% (cytoplasm reaction)	negative	+++, 100% (cytoplasm reaction)	negative
SALL4	+++, 100% (nuclear reaction)	negative	+++, 100% (nuclear reaction)	negative
OCT-4	++/+++, 70 % (nuclear reaction)	negative	++/+++, 70% (nuclear reaction)	++/+++, 40% (nuclear reaction)
WT1	negative	+, 40% (nuclear reaction)	negative	++, 80% (nuclear reaction)
Calretinin	negative	+/++, 30% (cytoplasm reaction)	negative	negative
InhibinB	negative	+/++, 40% (cytoplasm reaction)	negative	++/+++, 100% (cytoplasm reaction) +/++, 70% (cytoplasm reaction)
PLAP			++, 90% (membrane-cytoplasm reaction)	negative

CD117 – c-Kit, stem cell factor receptor, D2-40 – Podoplanin, SALL4 – Spalt-like transcription factor 4, WT1 – Wilms tumor 1, PLAP – placental-like alkaline phosphatase.

## Discussion

DSD are mainly classified according to Consensus statement of 2006, although some modifications and suggestions are already awaiting [1]. The incidence rate varies in 1:4500–1:5500 births [1,2,3]. DSD patients with Y chromosome material have a higher gonadal germ cell tumor risk. Sertoli and Leydig cell tumors are also more frequent in these patients [4].

Gonadal dysgenesis (GD) is a rare congenital condition considered to be the least clearly classified subgroup of DSD characterized by underdeveloped gonads due to chromosomal abnormalities or gene mutations [5,6]. Generally, three types of GD are distinguished: complete (CGD), characterized by female internal and external genitalia and bilateral streak-like gonads in individuals with 46,XX or 46,XY karyotypes; partial (PGD), identified in patients with 46,XY karyotype by ambiguous genitalia and bilateral dysgenetic testes or a dysgenetic testis and a streak-like gonad; or mixed (MGD), when various anatomical findings in internal and external genitalia are described in individuals with 45,X/46,XY mosaic karyotype or its variants [7]. In our case the presence of a dysgenetic testis and a streak-like gonad with 45,X/46,XY karyotype confirmed the diagnosis of mixed gonadal dysgenesis [8].

MGD is a rare condition characterized by various karyotype mosaicisms, dysgenetic gonads, and diverse anatomical findings in internal and external genitalia [7,8,9,10]. The incidence rate is 1–1,5 in10 000 newborns [1,6,11,12]. Mosaicism 45,X/46,XY is the most common karyotype found in patients diagnosed with MGD [10]. In general, MGD is associated with asymmetrical gonadal development [9]. As in the presented case, gonads are frequently a unilateral dysgenetic testis and a contralateral streak-like gonad with persistent asymmetrical Mullerian structures: underdeveloped uterus, fallopian tube ipsilateral to the streak-like gonad [6,13]. The broad range of phenotypical variations in MGD may be explained by the proportion of mosaic karyotype that is expressed in gonadal and other tissue cells [14,15]. Frequently Turner syndrome characteristics are found in individuals with 45,X/46,XY, the presence of 45,X cell line is associated with Turner syndrome fenotype [5,7,15,16]. Gonadal development and differentiation is determined by the predominant karyotype in gonadal tissue cells. It is suggested that gonosomal karyotype is the closest predictor of the patient’s sexual phenotype [17]. Therefore, a thorough clinical evaluation emphasizing genetic testing is very important [18]. Histological structure of the gonad cannot be predicted by only macroscopical features. Streak gonad is described as a flat fibrous tissue resembling underdeveloped gonad in the original place of an ovary without germ and supporting cells. Unfortunately, similar in the shape, gonads may contain germ cells and be the source of tumor. For that reason, the term “streak-like gonad“ was suggested and for the histological diagnosis of a pure streak, a dysgenetic gonad should be composed of only fibrous tissue devoid of germ and supporting cells [19,20]. Term dysgenesis have also been discussed by several authors as not precise and requiring specification in order to better predict the cancer and fertility possibilities [21]. Endocrinological evaluation is a necessary diagnostic step. High gonadotropins are particularly informative in cases of CGD [1,22]. In our case gonadotropins and testosterone at the “mini-puberty“ period and at 13 months may show normal testicular function. Further endocrinological follow-up is necessary in evaluating growth and approaching puberty later.

GD with the presence of the Y chromosome is associated with higher risk for germ cell neoplasia [4,6,13,18]. It is recommended to test two or more tissues when trying to detect Y chromosome sequences [17]. Specific probes for SRY detection can be used in order to evaluate the presence of the Y chromosome sequences [18]. Malignancy risk and timing of gonadectomy are one of the biggest challenges in GD patients [5]. Although germ cell tumor risk is the main reason for gonadectomy, surgery, especially irreversible, has to be based on multidisciplinary decision and focused on medical necessity [23]. Fertility preservation in DSD patients, although considered experimental, is investigated in specialized centers. Therefore, it should be kept in mind and discussed with family before deciding the sex of raring and gonadectomy [19]. In general, gonadal tumor risk in patients with MGD is 15–25 % and increases with age [5,6,7,12]. According to some authors, in patients with genital ambiguity and abdominal gonads this risk can rise up to 52% [9]. In order to prevent possible malignant transformation, preventive removal of gonads is recommended if female sex of rearing is chosen [12]. For streak-like gonads this should be done even if they seem morphologically not involved [6]. In mosaic 45,X/46,XY patients reared as males with scrotal testis, the risk for germ cell neoplasia is very low, but some cases are reported, therefore, regular examination after biopsy should be included in follow-up protocols [16]. Orchidopexy with biopsy of testicular part and resection of atypical, usually triangular, dysgenetic testicular outgrowth is advocated. The presence of a unilateral testis having the capacity for testosterone secretion prompts male sex of rearing as a common decision [9]. Leydig cell function may be evaluated by human chorionic gonadotrophin (hCG) stimulation test in undervirilized males [24]. In males, germ cell transformation into an obvious tumor tends to occur during or after puberty, therefore at least biopsy should be routinely recommended [6]. Gonadal biopsy is necessary for germ cell neoplasia risk assessment. Identification of specific histological characteristics provided valuable knowledge into germ cell neoplasia risk assessment, however, noninvasive screening markers for early-stage detection are lacking and yet to be implemented in clinical practice [19].

The most common tumors found in MGD are germ cell tumors [6,12]. Gonadoblastoma is the most common tumor in patients with mosaic 45,X/46,XY karyotype [12] It is a benign gonadal tumor that has high potential for malignant transformation, therefore, should be referred as in situ tumor, precursor for invasive germ cell neoplasia [12,17]. As reported, gonadoblastoma can occur in individuals as young as 3 months or even fetal life [7]. High gonadoblastoma risk is associated with particular gene mutations that have negative effects on Sertoli cell differentiation [5]. There are specific genes located in the (so-called) gonadoblastoma locus on the Y chromosome that are associated with the development of germ cell neoplasia [4]. Higher risk of malignant transformation is associated with the octamer binding transcription factor 3/4 (OCT3/4) and testis-specific protein on the Y chromosome (*TSPY*) [12,19]. Combining OCT3/4 and *TSPY* markers may increase the chance of detecting neoplastic germ cells in dysgenetic gonads. Although, positive OCT3/4 in patients younger than 2 years old suggests delayed maturation, while placental-like alkaline phosphatase (PLAP) and stem cell factor receptor (c-Kit – CD117) staining may be demonstrated in normal boys undergoing puberty [11,25]. Therefore, diagnosis of gonadoblastoma relies on complex evaluation.

In summary, multidisciplinary approach with involvement of parents and, if eligible, patients is a necessary requirement when managing DSD patients. In many cases DSD is recognized on the first objective evaluation. It should be followed by accurate history and physical examination [1]. Guidelines for further investigation until the correct diagnosis have been proposed [22]. The necessary final step of evaluation, gonadectomy of the streak-like gonad and biopsy or gonadectomy of dysgenetic testicle should be performed at an early age. Our review is limited due to relatively small number of studies, particularly large scale and prospective.
